# Restoration of Reflection Spectra in a Serial FBG Sensor Array of a WDM/TDM Measurement System

**DOI:** 10.3390/s120912836

**Published:** 2012-09-20

**Authors:** Dusun Hwang, Dae-Cheol Seo, Il-Bum Kwon, Youngjoo Chung

**Affiliations:** 1 Center for Safety Measurement, Korea Research Institute of Standards and Science, Yuseong-gu, Daejeon 305-340, Korea; E-Mails: lobe2sun@naver.com (D.H.); dcseo@kriss.re.kr (D.-C.S.); 2 Department of Information and Communications, School of Photon Science and Technology, Gwangju Institute of Science and Technology, 1 Oryong-dong, Buk-gu, Gwangju 500-712, Korea; E-Mail: ychung@gist.ac.kr

**Keywords:** reflection spectrum restoration method, fiber Bragg grating sensor array, WDM/TDM, maximum peak detection error, strain experiment

## Abstract

A restoration method for reflection spectra in a serial FBG sensor array with spectral shadowing is proposed and experimentally demonstrated in a WDM/TDM combined multiplexing system. The SNR of each FBG sensor is formulated and analyzed as a function of the number and reflectivities of serial FBG sensors. The maximum number of FBG sensors in a single fiber line can be determined by the approximate formula. In the test using two FBG sensors, the restored reflection spectrum of second FBG sensor is shown to be very well matched with the original reflection spectrum. Using the proposed restoration method, the maximum peak detection error in a strain experiment is suppressed drastically by almost seven-fold, from 0.074 nm to 0.011 nm.

## 1. Introduction

Fiber Bragg gratings (FBGs) have many advantages like immunity to electromagnetic interference, integration into composite materials without degradation of the strength of the host material, capacity for multiplexing many sensors in a single fiber lead, and capacity for mass production with good repeatability, making them potentially competitive against conventional electrical strain sensors. Many researchers have studied the use of FBG sensors to measure various parameters, such as strain [1–3], acceleration [4,5], temperature [6], humidity [7], *etc*. With their wavelength-encoded nature and the capacity for wavelength division multiplexing (WDM), FBGs have been demonstrated for a number of sensing applications, especially for strain measurements for monitoring structural health status in smart structures [8]. A primary advantage of FBGs for distributed sensing is that multiple sensors can be interrogated along a single fiber. In the WDM technique, it is important to detect the accurate Bragg wavelengths of the FBGs within the sensor network because the Bragg wavelengths are directly related with the measuring parameters such as strain or temperature. There is one key issue to establish the accurate detection of Bragg wavelengths when the spectra of the FBGs within the network are partially or fully overlapped. A simulated annealing technique is used to determine the accurate Bragg wavelengths of the FBGs in the sensor network [9]. Also, several numerical processing techniques such as minimum variance shift technique [10], genetic algorithm [11], improved differential evolution algorithm [12], can be applied to a measured spectrum to detect the Bragg wavelengths of FBGs in the sensor network. However, the number of FBGs in WDM is limited by the wavelength range of the broadband light source of the sensor system. Therefore, WDM/TDM (time division multiplexing) hybrid techniques can be used to overcome the limitations of WDM and to extend the maximum number of sensors that can be supported by WDM. Maximizing the number of sensors is very important for large-scale structural health monitoring which requires a huge number of sensor heads. Because these hybrid techniques basically restore the spectra of FBGs, these techniques don't use numerical techniques for the accurate Bragg wavelength detection except for noise rejection schemes such as noise filtering, curve fitting, *etc*. The WDM/TDM hybrid approach has been demonstrated using a 3 × 3 grating array with time-gating at the detector combined with wavelength detection using the scanning Fabry Perot (FP) filter [13]. This scheme has two problems: multiple-reflection [14] and spectral-shadowing [8]. Although the change of topology from serial to parallel network or branching network can be employed to avoid these effects, the serial connection of FBGs is most preferred because of its cost effectiveness. Recently several techniques for improvement of WDM/TDM combined systems with serial FBG sensor arrays were proposed. To reduce the scanning time, the optical code division multiplexing (CDM) technique was proposed [15] and ring cavity techniques are proposed for enhancement of SNR and extraction of specific TDM groups [16,17]. Frequency domain multiplexing techniques have been also demonstrated [18]. Even in these modified methods the spectral shadowing effect was not eliminated and this contributes to errors in peak detection and peak power measurement. The multiple reflection effect between FBGs with almost same wavelengths is strongly dependent on the reflectivity of the grating and can be minimized by the use of gratings with low reflectivities because it affects predominantly only three neighboring FBGs. However, the spectral-shadowing effect cannot be completely eliminated because this is a cumulative effect and the upstream FBGs affect the reflection spectra of the downstream FBGs. Thus, if the number of FBGs is increased, this shadowing effect becomes the dominant source of spectrum distortion. In WDM/TDM sensor array, the spectral shadowing effect causes larger error in peak detection as the number of sensors is increased. In this work, we will propose and experimentally demonstrate a new spectrum restoration method that reduces the peak detection error due to the shadowing effect in WDM/TDM serial sensor arrays.

## 2. Theory and Simulation

**Figure 1.**
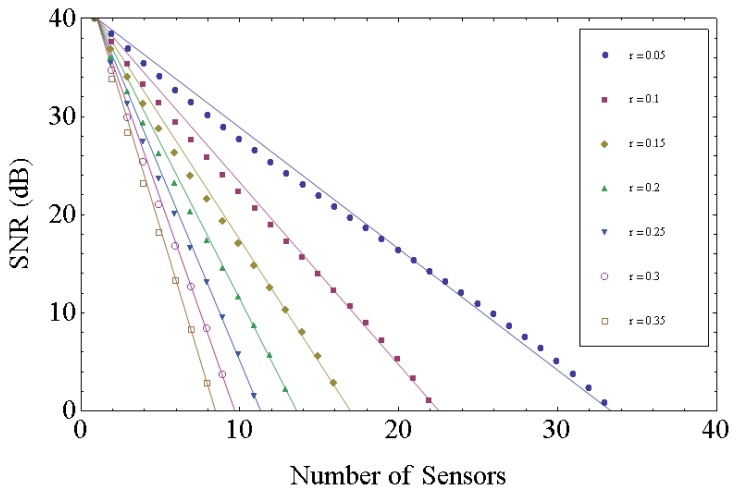
SNR degradation due to the increase of number of FBG sensors.

In an ideal serial FBG array with negligible splicing loss and attenuation, the effective reflectivity with the shadowing effect can be expressed as:

(1) $${R_{n}}^{\text{eff}}(\lambda )=\overset{n-1}{\underset{k=1}{\Pi }}{\left(1-R_{k}(\lambda )\right)}^{2}R_{n}(\lambda )$$

where *R_n_^eff^*(*λ*) is the effective reflectivity of *n*-th FBG and *R_k_*(*λ*) is the reflectivity of the *k*-th FBG. Since the reflectivity of each FBG cannot be determined from the reflected optical signals having shadowing effect on that reflected spectra, we rewrite the above Equation in terms of the detected optical power as:

(2) $$R_{n}(\lambda )=(P_{n}(\lambda )-P_{\text{ref}}(\lambda ))/\{({P_{0}}^{AC}(\lambda )-{P_{0}}^{DC}(\lambda ))\overset{n-1}{\underset{k=1}{\Pi }}\alpha_{k}(\lambda ){\left(1-R_{k}(\lambda )\right)}^{2}\}$$

where *P_n_*(*λ*) is the detected reflection optical power from *n*-th FBG, *P_ref_*(*λ*) is the reference level due to the electro-optic modulator (EOM) leakage, *P*_0_*^AC^*(*λ*) is the AC component of incident optical power, *P*_0_*^DC^*(*λ*) is the DC component of incident optical power due to the EOM leakage, and *α_k_*(*λ*) is the loss between the *k* − 1 -th FBG and the *k*-th FBG including splicing loss and attenuation. We can calculate the reflectivity of the *n*-th FBG using Equation (2) recurrently. In Equation (2) the order of *P_0_* is increased by *2n* if the number of sensors *n* is increased by 1. This rapid increase makes the proposed restoration algorithm numerically unstable for a large number *n*. In other words, small amount of noise in the input power *P_0_* or reflection spectra of upstream FBGs can make large deformation of downstream FBG spectra. The SNR of reflection spectrum is degraded as the number of sensors *n* is increased. For the case of exactly overlapped spectra, the SNR can be calculated by the following equation:

(3) $$\begin{array}{cc} \text{SNR}(n) & =10\log_{10}\left(\frac{r}{R_{n}^{\text{res}}-r}\right),\\ R_{n}^{\text{res}} & =\left(r{\left(1-r\right)}^{2n-1}+\frac{r}{s}\right)/\prod_{k=1}^{n-1}{\left(1-R_{k}^{\text{res}}\right)}^{2} \end{array}$$

where the SNR(*n*) is the SNR of the reflection spectrum of the *n*-th FBG, *r* is the actual reflectivity of each FBG, $R_{n}^{\text{res}}$ is the restored reflectivity, and *s* is the linear scaled SNR of reflection from each FBG without the shadowing effect. This value *s* will be cumulated as *n* is increased and will degrade the SNR. The SNR degradation due to the increase of number of FBG sensors can be calculated as shown in Figure 1.

From the calculated data, we formulated the SNR as a function of number of sensors and reflectivity of each sensor as follows:

(4) SNR(n,r)=SNR(1)−5.21(n−1)(r+0.00643)(r+2.1286)

where SNR(*n*, *r*) is the SNR of the reflection of the *n*-th FBG, SNR (1) is the SNR of the reflection of the first FBG, and *r* is the reflectivity of the FBGs. The lines in Figure 1 show the result of this linear fitting. The maximum standard deviation of SNR between the calculated data from Equation (3) and the linear fitting from Equation (4) is 0.23 dB at the case of r = 0.05. If the performance of interrogation system is measured, our formula can estimate the maximum number of sensors that the system can support.

For example, in our experiment we used two FBGs which have about 60% reflectivity and confirmed the spectrum restoration. Using our SNR analysis, the SNR degradation of our system coincides with the SNR degradation of 14 FBGs with 5% of reflectivity. Thus using our spectrum restoration method, it would be possible to interrogate 140 FBG sensors with successfully suppressed spectral shadowing effect if the FBGs with 5% of reflectivity are wavelength-division-multiplexed into 10 groups. If one FBG has a much larger reflectivity than the others, then the SNR of the total sensor system and the multiple reflections of same FBGs will occur. In that case, our proposed theory will not be applied on that system.

## 3. Experiments

**Figure 2.**
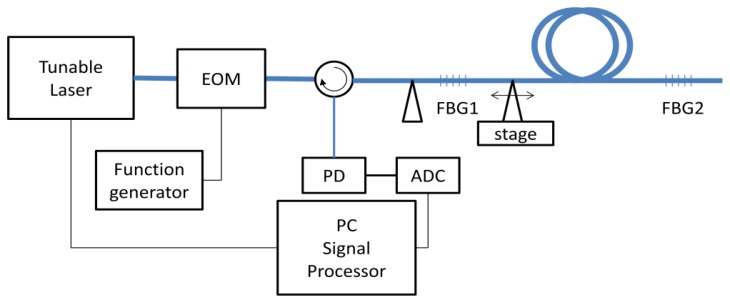
Experimental setup for investigation of shadowing effect of FBG sensor array in WDM/TDM multiplexing.

**Figure 3.**
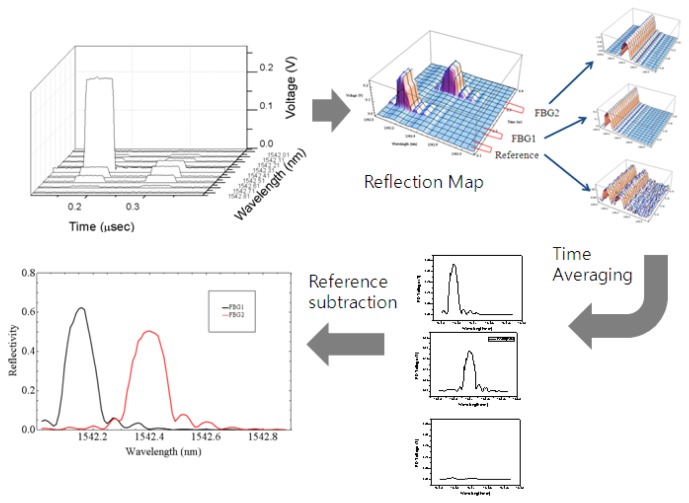
Signal processing procedure to get the each reflection spectrum from the WDM/TDM signal.

Figure 2 shows our experimental setup. A computer-controlled tunable laser source (TLS, Ando AQ4321B) with 200 kHz linewidth was connected to an electro-optic modulator (EOM, JDSU). The pulse pattern generator (HP 81110A) generated pulses between −1 V and 3 V with 50 ns of duration and 500 micro-second of period. The voltage level of pulse was adjusted so that the extinction ratio of EOM was maximized. The pulsed laser from EOM was launched into the FBGs passing through the circulator. The reflected beam from FBGs returned to the photodiode (New Focus 1811). Using an oscilloscope (Tektronics TDS3034), we measured the reflection from the FBGs in time domain with 500 MHz of sampling rate as the TLS was swept from 1,542 nm to 1,543 nm with 0.01 nm steps. The data was gathered using a computer together with the wavelength data and the WDM/TDM maps of sensors could be obtained as shown in Figure 3. The TLS and oscilloscope were simultaneously controlled by the computer.

FBG1 was attached on the linear stage and the center wavelength was changed by applying strain on it. The initial center wavelengths were 1,542.16 nm and 1,542.4 nm for FBG1 and FBG2, respectively. We measured the reflection map while changing the applied strain on the FBG1 from 0 to 480 micro-strain, which is equivalent to the wavelength range from 1,542.16 to 1,542.67 nm because the strain sensitivity of the FBG1 was 1.08 nm/micro-strain. From the acquired reflection map, we extracted the reflection spectrum of each FBG and reference by time averaging. In this configuration the reference spectrum was obtained from the leakage beam from EOM. We calibrated the reflection spectra of FBGs by subtracting the reference level. The details of signal processing procedure are shown in Figure 3.

**Figure 4.**
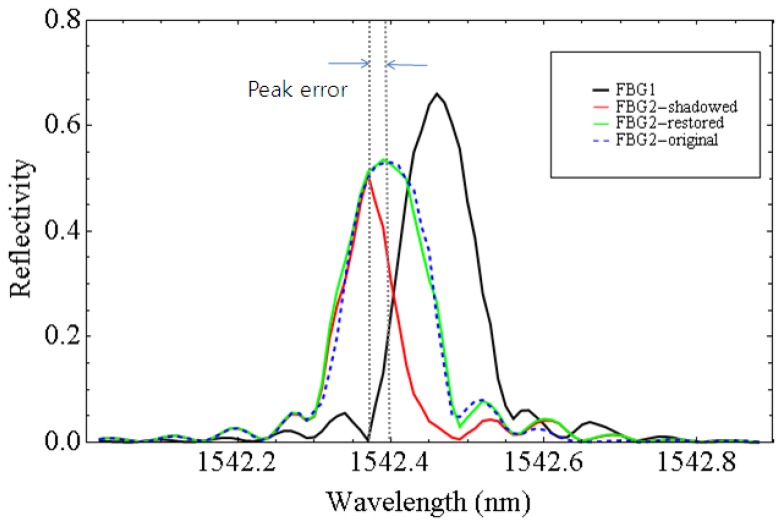
Comparison of the spectra between FBG1 and FBG2.

We acquired the spectra of the FBG1 and the shadowed FBG2 after the signal processing described in Figure 3. The reflection spectra were obtained by sweeping the wavelengths in the time domain. We found the peak location of each spectrum by Gaussian fitting. Even though FBG2 was fixed, the distortion due to the shadowing effect caused the peak wavelength of FBG2 to shift away from the peak wavelength of FBG1. Using Equation (3) given in the previous section, we restored the reflection spectrum of FBG2 as shown in Figure 4. The blue dashed curve indicates the original reflection spectrum of FBG2 with little shadowing effect due to FBG1 when the center wavelength of FBG1 was at 1,542.66 nm. When FBG1's reflection spectrum moved closer to FBG2's (the black curve), the reflection spectrum of FBG2 became distorted (the red curve). Using our restoration Equation (3), the reflection spectrum of the shadowed FBG2 was successfully restored (the green curve), which is nearly the same as the original blue dashed curve. The restoration equation is applied for each wavelength while sweeping the wavelengths and the reflection spectrum was restored concurrently in real-time.

**Figure 5.**
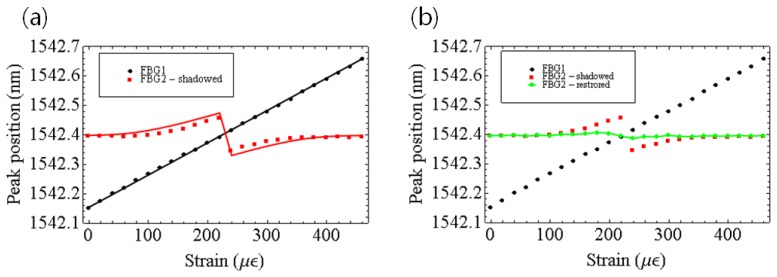
(**a**) The wavelength detection error due to the shadowing effect (dots: experimental data, solid curve: simulation data); (**b**) suppression of the shadowing effect using the restoration method.

The data we need in sensor applications are usually the peak wavelengths of FBGs. To this end, we fit our experimental results to the Gaussian model to get the peak wavelengths of FBGs. The change of the peak wavelengths of FBGs due to the strain variation are shown in Figure 5. Figure 5(a) shows the measured results (dots) and the simulation results (lines) before restoration. The maximum difference between the two was about 0.017 nm. The maximum error of 0.074 nm occurred when the peak wavelengths of FBG1 and FBG2 nearly overlapped. Figure 5(b) shows the suppression of peak detection error after the spectrum restoration. The maximum peak detection error was reduced from 0.074 nm to 0.011 nm by means of the spectrum restoration. The accuracy was improved by almost 7 times. Even this restoration spectrum has sufficient SNR and normal spectrum shapes, in real field situation, the FBG spectrum usually has low SNR and un-normal spectrum shapes. In this kind of real noisy measured spectrum, it is to be very useful to apply some numerical techniques, proposed in references [9–12], to detect the accurate Bragg grating wavelengths of FBGs.

## 4. Conclusions

We have proposed and experimentally demonstrated a reflection spectrum restoration method for serial FBG array and performed the SNR analysis of this method. It was shown that the shadowing effect of serial FBG array could be successfully suppressed using the proposed method and the maximum peak wavelength error was reduced by almost seven times (0.074 nm → 0.011 nm). Since this method is a comprehensive spectrum restoration method for spectral shadowing effect, it is applicable not only to our basic WDM/TDM system, but also to any modified WDM/TDM systems with a serial FBG topology. The SNR approximation formula will be also useful for the design of various serial FBG sensor systems.

## References

[1] Lima H.F., Antunes P.F., de Lemos Pinto J., Nogueira R.N. (2010). Simultaneous measurement of strain and temperature with a single fiber Bragg grating written in a tapered optical fiber. IEEE Sens. J..

[2] Ma C.C., Wang C.W. (2009). Transient strain measurements of a suspended cable under impact loadings using fiber bragg grating sensors. IEEE Sens. J..

[3] Zhang Z., Yan L., Pan W., Luo B., Wang P., Guo L., Zhou W. (2012). Sensitivity enhancement of strain sensing utilizing a differential pair of fiber bragg gratings. Sensors.

[4] Tsuda H. (2010). Fiber Bragg grating vibration-sensing system, insensitive to Bragg wavelength and employing fiber ring laser. Opt. Lett..

[5] Antunes P.F.C., Marques C.A., Varum H., Andre P.S. (2012). Biaxial optical accelerometer and high-angle inclinometer with temperature and cross-axis insensitivity. IEEE Sens. J..

[6] Park H., Song M. (2008). Linear FBG temperature sensor interrogation with fabry-perot ITU multi-wavelength reference. Sensors.

[7] Correia S.F.H., Antunes P., Pecoraro E., Lima P.P., Varum H., Carlos L.D., Ferreira R.A.S., André P.S. (2012). Optical fiber relative humidity sensor based on a FBG with a Di-ureasil coating. Sensors.

[8] Kersey A.D., Davis M.A., Patrick H.J., LeBlanc M., Koo K.P., Askins C.G., Putnam M.A., Friebele E.J. (1997). Fiber grating sensors. J. Lightwave Technol..

[9] Shi C.Z., Zeng N., Chan C.C., Liao Y.B., Jin W., Zhang L. (2004). Improving the performance of FBG sensors in a WDM network using a simulated annealing technique. IEEE Photonics Technol. Lett..

[10] Chan C.C., Shi C.Z., Gong J.M., Jin W., Demokan M.S. (2003). Enhancement of the measurement range of FBG sensors in a WDM network using a minimum variance shift technique coupled with amplitude-wavelength dual coding. Opt. Commun..

[11] Li P., Zhao X. Increasing the Number of Series FBG Sensors in WDM Network Using a Genetic Algorithm.

[12] Liu D., Tang K., Yang Z., Liu D. (2011). A fiber Bragg grating sensor network using an improved differential evolution algorithm. IEEE Photonics Technol. Lett..

[13] Berkoff T.A., Davis M.A., Bellemore D.G., Kersey A.D., Williams G.M., Putnam M.A. (1995). Hybrid Time- and Wavelength-Division Multiplexed Fiber Bragg Grating Sensor Array. Proc. SPIE.

[14] Kersey A.D., Dorsey K.L., Dandridge A. (1989). Cross talk in a fiber-optic Fabry-Perot sensor array with ring reflectors. Opt. Lett..

[15] Abbenseth S., Lochmann S.I. (2005). Distinct enlargement of network size or measurement speed for serial FBG sensor networks utilizing SIK-DS-CDMA. J. Phys.: Conf. Ser..

[16] Chung W.H., Hwa-Yaw T., Wai P.K.A., Khandelwal A. (2005). Time- and wavelength-division multiplexing of FBG sensors using a semiconductor optical amplifier in ring cavity configuration. IEEE Photonics Technol. Lett..

[17] Chen D., Shu C., He S. (2008). Multiple fiber Bragg grating interrogation based on a spectrum-limited Fourier domain mode-locking fiber laser. Opt. Lett..

[18] Zhou B., Guan Z., Yan C., He S. (2008). Interrogation technique for a fiber Bragg grating sensing array based on a Sagnac interferometer and an acousto-optic modulator. Opt. Lett..

